# Effectiveness of a nurse-led phone intervention on body composition in adults with prediabetes and overweight/obesity: PREDIPHONE secondary analysis

**DOI:** 10.3389/fendo.2026.1911675

**Published:** 2026-09-17

**Authors:** Natalia Garcia-Coll, María Arias-Fernández, Manuela Abbate, Marina Torres-Carballo, Aina Huguet-Torres, Rocío Zamanillo-Campos, Ignacio Ricci-Cabello, Miquel Bennasar-Veny, Aina Maria Yañez, Aina M Galmes-Panades

**Affiliations:** 1 Research Group on Global Health, University of Balearic Islands, Palma, Spain; 2 Research Group on Nursing, Community and Global Health, Health Research Institute of the Balearic Islands (IdISBa), Palma, Spain; 3 Nursing and Physiotherapy Department, University of the Balearic Islands, Palma, Spain; 4 Primary Care of Mallorca, Public Health Service of the Balearic Islands (Ib-Salut), Palma, Spain; 5 Network for Research on Chronicity, Primary Care, and Health Promotion (RICAPPS), Health Research Institute of the Balearic Islands (IdISBa), Palma, Spain; 6 Research Group on Primary Care, Health Services and Evidence-Based Medicine (EVISAP), Health Research Institute of the Balearic Islands (IdISBa), Palma, Illes Balears, Spain; 7 Primary Care Research Unit of Mallorca, Balearic Islands Health Service, Palma, Illes Balears, Spain; 8 Centre for Biomedical Research Network (CIBER) in Epidemiology and Public Health (CIBERESP), Instituto de Salud Carlos III, Madrid, Spain; 9 Physical Activity and Sport Sciences Research Group (GICAFE), Institute for Educational Research and Innovation (IRIE), University of the Balearic Islands, Palma, Spain; 10 Centre for Biomedical Research Network (CIBER) of Physiopathology of Obesity and Nutrition (CIBEROBN), Instituto de Salud Carlos III, Madrid, Spain

**Keywords:** body composition, Mediterranean diet, obesity, physical activity, prediabetes, telephone intervention, visceral adipose tissue

## Abstract

**Background:**

Prediabetes and obesity are strongly linked to metabolic risk, with visceral adipose tissue playing a central role. Lifestyle interventions combining physical activity and healthy diet are effective for body composition improvement, but most are resource-intensive. Evidence on low-intensity, scalable approaches delivered by primary care professionals, such as nurse-led telephone interventions, remains limited. This secondary analysis of the PREDIPHONE randomized controlled trial assessed the effectiveness of a low-intensity nurse-led telephone lifestyle intervention on body composition outcomes at 4 months (mid-intervention), 9 months (end of intervention), and 15 months (6 months post-intervention).

**Methods:**

The analysis included 174 participants with available body composition data originally enrolled in the PREDIPHONE trial. Original group allocation was retained. Body composition parameters (fat mass, muscle mass, and visceral adipose tissue) were measured using bioelectrical impedance analysis at each time point. Physical activity and adherence to the Mediterranean diet were assessed using validated questionnaires. Group differences were evaluated using general linear models adjusted for baseline values, age, and sex.

**Results:**

Participants had a mean age of 61.1 years, and 52.3% were women. Compared with the active SMS-based control group, the intervention group achieved short-term reductions in visceral adipose tissue and fat mass at 4 months. These changes were not maintained at 9 or 15 months. No significant differences were observed in muscle mass or body mass index.

**Conclusion:**

In this population, a low-intensity, nurse-led telephone intervention produced modest short-term improvements in body composition without sustained effects. More intensive or prolonged strategies may be required to produce clinically meaningful and sustainable changes in primary care settings.

**Trial registration:**

Clinicaltrials.gov, identifier NCT04735640.

## Introduction

Obesity is a complex, multifactorial, and chronic condition that has reached pandemic proportions worldwide, with prevalence continuing to rise in both global and national contexts, including Spain (1–3). Overweight and obesity are major drivers of insulin resistance and key contributors to the development of type 2 diabetes (T2D) (4). Importantly, clinically significant weight loss has been shown to improve haemoglobin A1c (HbA1c), and fasting plasma glucose (FPG) levels and is associated with a greater likelihood of remission of T2D, with larger weight losses associated with a higher likelihood of remission (5).

Beyond total body weight, body composition plays a central role in metabolic health. In particular, excessive accumulation of visceral adipose tissue (VAT) is strongly associated with insulin resistance, hyperglycemia (6), and altered blood lipid profiles, and contributes to the progression from normoglycemia to prediabetes and T2D (4). Conversely, reductions in VAT and preservation or increases in lean mass are associated with improved metabolic outcomes (7).

Lifestyle interventions that integrate structured physical activity (PA) and dietary modifications have been recognized as effective strategies for body composition modification and metabolic health (8). Regular PA improves insulin sensitivity, reduces VAT and total body fat, regulates blood glucose and reduces the risk of cardiovascular disease in people at risk of T2D (9, 10). High-quality dietary patterns, such as the Mediterranean Diet (MedDiet), characterized by higher intakes of fruits, vegetables, whole grains, and healthy fats, have been shown to improve body composition and prevent T2D, independently of weight loss (11). However, despite their efficacy, most lifestyle interventions evaluated to date are intensive and resource-demanding, which constrains their scalability and implementation in routine primary care (12, 13).

Furthermore, although many studies report improvements in glycemic control or weight, relatively few specifically evaluate changes in VAT or muscle mass, despite their clinical relevance (12). Evidence is also scarce regarding the long-term effectiveness of low-intensity, scalable lifestyle interventions, particularly those delivered by primary care professionals through accessible modalities such as telephone-based support. Such approaches may offer a pragmatic and cost-effective strategy to extend the reach of preventive interventions while maintaining effectiveness.

The present study is a secondary analysis of the PREDIPHONE randomized controlled trial aimed to evaluate the effectiveness of a low-intensity, nurse-led personalized telephone lifestyle intervention compared to SMS-based lifestyle advice in people with prediabetes and overweight or obesity, assessing changes in body composition at 4 months, at intervention completion (9 months), and at 6 months post-intervention (15 months).

## Methods

### Study design

The PREDIPHONE study was a randomized controlled clinical trial with two parallel groups, registered at Clinicaltrials.gov (NCT04735640) (14). The primary outcome of the trial was change in fasting plasma glucose, and the main results have been reported elsewhere (15).

The present study is a secondary analysis of the PREDIPHONE trial and focuses on body composition outcomes. The primary aim of the analysis was not to characterize the longitudinal trajectory of body-composition changes or to test a treatment-by-time interaction, but to estimate the between-group intervention effect at the prespecified follow-up assessment(s). Therefore, baseline-adjusted GLMs were used to estimate the intervention effect at each assessment in the original units of the outcome.

### Study setting and sampling

The study was conducted in five primary care centers located in Mallorca, Balearic Islands, Spain. A total of 206 participants were randomized to either the intervention (n=103) or control (n=103) group. For the present analysis, we included participants with available body composition data at baseline, resulting in an analytical sample of 174 individuals. Original group allocation was retained.

### Inclusion and exclusion criteria

Eligible participants were women and men aged between 25 and 75 years, with a BMI between 27 and 40 kg/m^2^, and prediabetes [FPG levels between 100 and 125 mg/dL, according to the American Diabetes Association (16)]. Exclusion criteria included a prior diagnosis of T2D, current antidiabetic treatment, FPG ≥126 mg/dL, or systemic glucocorticoid use. The full list of exclusion criteria can be found in the study protocol (14).

### Study intervention

A detailed description of the telephone-based intervention and SMS-based advice is reported in the study protocol (14). Briefly, participants in the intervention group (IG) received personalized advice on healthy diet and PA for 9 months. The intervention included an initial face-to-face session followed by 8-12 telephone consultations during follow-up. Participants in the control group (CG) received between 4 and 5 automated SMS per week (for a total of 150 SMS) for 9 months. The SMS, developed by a multidisciplinary team, provided general advice on PA and healthy diet and were sent automatically by the Balearic Islands Health Service.

### Data collection and measurements

With the exception of sociodemographic data, medical history, medication use, smoking status and height (m), which were collected at baseline only, data were collected at baseline, at 4 months (mid-intervention), at 9 months (intervention completion) and at 15 months (6 months post-intervention).

Participants were categorized based on their self-reported occupation as either white-collar (executives, managers, university professionals, intermediate workers, and employees) or blue-collar (manual workers) (17). Smoking status was classified as never, former or current smoker, following the WHO classification (18).

Anthropometric measurements were obtained according to the International Standards for Anthropometric Assessment (19). Body weight and composition (total body fat percentage, muscle mass [kg], and VAT [measured on a scale from 1 to 59]) were assessed using a tetrapolar bioelectrical impedance analysis (BIA) scale (Tanita MC-780, Tanita Corp, Tokyo, Japan), with weight recorded to the nearest 0.1 kg (20). Participants were asked to wear light clothing and stand barefoot on the metal contact pads during measurement. Height was measured to the nearest 0.5 cm using a stadiometer (Seca 220 (CM) Telescopic Height Rod for Column Scales, Seca GmbH, Hamburg, Germany) with participants standing upright and their heads in the anatomical position. BMI was calculated as weight (kg)/height (m²), with overweight defined as BMI between 27 and 29.9, and obesity defined as BMI ≥ 30 kg/m². Body composition is closely associated with metabolic health (6); therefore, measuring it provides an indication of an individual’s metabolic health. Tetrapolar bioimpedance is a portable, safe and non-invasive method.

Waist circumference (WC) was measured twice at the midpoint between the last rib and the iliac crest using an anthropometric tape. Participants stood upright with arms crossed over their chests and feet evenly spaced. The mean of the two measurements was used for statistical analyses. Abdominal obesity was defined as WC ≥ 88 cm in women, and ≥ 102 cm in men (21).

Blood pressure (BP) was measured using an automated sphygmomanometer (OMRON M3, Healthcare Europe, Barcelona, Spain) with participants seated after 15 minutes of rest. Two measurements were taken one minute apart, and the average was recorded (22). The arm with the highest BP values was used for statistical analysis.

Blood samples were collected from the antecubital vein using appropriate vacutainers after an 8-12 hour overnight fast for the analysis of FPG, HbA1c, total cholesterol, high-density lipoprotein cholesterol (HDL-c), triglycerides (TG) (measured in serum using the Abbott ARCHITECT c16000 analyzer (Abbott Laboratories, Abbott Park, IL, USA) with specific commercial kits) and low-density lipoprotein cholesterol (LDL-c) (calculated using the Friedewald formula). Analyses were conducted at Son Espases University Hospital, Majorca, Balearic Islands, Spain.

Physical activity levels were assessed using the validated self-reported REGICOR PA questionnaire for adults, which assesses PA levels in leisure time over the preceding 12 months (23). The REGICOR PA questionnaire provides data on the average daily energy expenditure from PA, expressed as metabolic equivalents (MET’s) (24), including data on the average monthly duration of sedentary (0-1.5 MET), light (1.5-3 MET), moderate (3-5.9 MET), and vigorous (≥6 MET) PA. The total weekly hours spent in sedentary behavior (SB) were also collected. Additionally, selected questions related to SB were included from the validated Spanish version of the Nurses’ Health Study (NHS) questionnaire (25). The NHS questionnaire specifically captures the average daily hours spent on sedentary activities, defining SB as a sitting time of ≥7 hours per day on average.

Diet quality, and adherence to dietary intervention were assessed using the 14-item MedDiet adherence questionnaire (26). Each item was scored as 1 (compliance) or 0 (non-compliance) with a total score ranging from 0 to 14, where 0 indicated no adherence and 14 represented maximum adherence. A score of ≤7 was considered low adherence, while a score of ≥10 was classified as high adherence.

### Outcomes

Body composition outcomes, including VAT, muscle mass (kg), body fat mass (kg), BMI and WC, were assessed at 4 months (mid-intervention), at 9 months (intervention completion) and at 15 months (6 months post-intervention).

Physical activity and adherence to the MedDiet were considered as intermediate behavioral outcomes.

### Data analysis

This analysis followed an available-case approach and included participants with available data on VAT at baseline (n=174), 4 months (n=170), 9 months (n=174), and 15 months (n=161).

Variable distributions were assessed by the Kolmogorov-Smirnov test. Continuous variables are presented as mean and standard deviation (SD), and categorical variables are presented as counts and percentages. Baseline differences between groups were assessed using Student’s t-test for continuous variables and the chi-square test for categorical variables. Changes in body composition outcomes were analyzed at each time point using a general linear model (GLM), with group (intervention vs control) as the main independent variable, adjusting for baseline values of the outcome, age and sex. Therefore, baseline-adjusted GLMs were used to estimate the intervention effect at each assessment in the original units of the outcome.

Additionally, stratified analysis by sex was also performed using GLM. All analyses were conducted by a statistician blinded to group assignment and independently verified by a second researcher. A data safety monitoring committee oversaw the study.

Prior to conducting the GLM analyses, normality of all study variables was assessed using the Kolmogorov-Smirnov test, and data were examined for potential outliers or extreme values. No substantial departures from normality or influential values were identified. Homoscedasticity was assessed by examining the residuals from the fitted models, and no relevant violations were detected. Missing data were handled using an available-case approach, including all participants with available data at each time point. No additional sensitivity analyses were performed.

Statistical significance was defined as a p-value <0.05, and 95% confidence intervals (CI) were reported. All analyses were performed using Stata v.16 (StataCorp, Texas, USA).

## Results

### Participants

Of the 206 participants randomized in the PREDIPHONE study, 174 were included in this secondary analysis due to the availability of body composition data (IG n=84; CG n=90). Follow-up data were available for 170 participants (IG: n=82; CG: n=88) at 4 months, 174 (IG: n=84; CG: n=90) at 9 months and 161 (IG: n=80; CG: n=81) at 15 months. Four participants without available body composition data at 4 months returned and provided valid measurements at 9 months. The study flowchart is shown in **Figure 1**.

**Figure 1:**
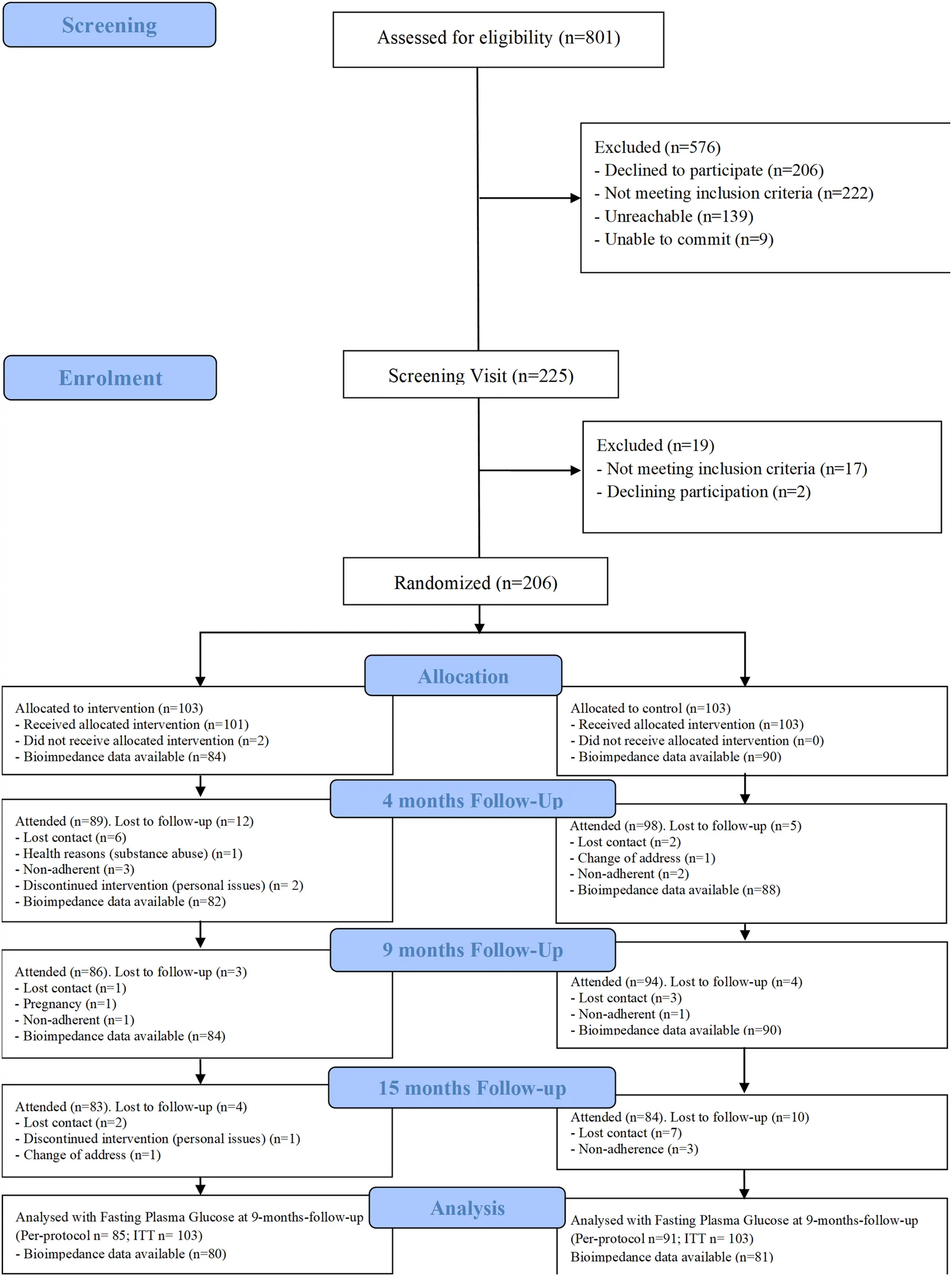
Flowchart of the study sample.

### Baseline characteristics

**Table 1** shows baseline characteristics of the overall sample and by group. Mean age was 61.10 years (SD 8.90) and 52.30% were women. Mean BMI was 32.17 kg/m^2^ (SD 3.46) and mean WC was 105.10 cm (SD 10.32), with 85.63% of participants presenting abdominal obesity. Baseline characteristics were compared between participants who completed the 15-month follow-up visit and those who withdrew from the study prior to the final assessment, in order to identify potential differences between the two groups. As shown in **Supplementary Table 1**, no substantial baseline differences were observed between participants who completed the study and those who dropped out (n=13), suggesting that attrition was unlikely to have introduced a systematic bias in the study sample.

**Table 1:** Characteristics of participants at baseline.

Variables	Total (n=174)	Control (n=90)	Intervention (n=84)
**Age**	61.10 (8.90)	61.29 (9.77)	60.90 (7.91)
**Woman (n, [%])**	91 (52.30)	46 (50.55)	45 (49.45)
Social class^1^ (n, [%]):			
White collar	36 (20.69)	17 (18.89)	19 (22.62)
Blue collar	138 (79.31)	73 (81.11)	65 (77.38)
Education level (n, [%]):			
University degree	29 (16.67)	12 (13.33)	17 (20.24)
Higher education	62 (35.63)	25 (27.78)	37 (44.05)
Basic studies	83 (47.70)	53 (58.89)	30 (35.71)
Smoking habits^2^ (n, [%]):			
Never	77 (44.25)	39 (43.33)	38 (45.24)
Current	22 (12.64)	13 (14.44)	9 (10.71)
Former	75 (43.10)	38 (42.22)	37 (44.05)
Anthropometric measures:			
BMI (kg/m^2^)	32.17 (3.46)	32.33 (3.69)	32.01 (3.22)
WC (cm)	105.10 (10.32)	105.90 (9.80)	104.25 (10.84)
Abdominal obesity^a^			
No	25 (14.37)	16 (17.78)	9 (10.71)
Yes	149 (85.63)	74 (82.22)	75 (89.29)
Body composition:			
VAT (units)	13.50 (3.94)	13.50 (3.61)	13.33 (4.29)
Body fat mass (kg)	30.71 (7.96)	30.92 (9.02)	30.49 (6.70)
Body fat mass (%)	36.00 (6.96)	36.09 (8.02)	35.90 (5.64)
Muscle mass (kg)	54.63 (11.32)	54.34 (10.23)	54.93 (12.45)
Clinical parameters:			
SBP (mmHg)	134.92 (14.65)	135.58 (14.54)	134.20 (14.81)
DBP (mmHg)	84.05 (9.31)	83.77 (9.52)	84.36 (9.12)
HbA1c (%) (n=135)	5.90 (0.33)	5.90 (0.36)	5.90 (0.30)
FPG (mg/dl)	108.98 (6.47)	109.19 (6.33)	108.75 (6.65)
Total cholesterol (mg/dl) (n=168)	195.45 (34.95)	193.78 (33.77)	197.21 (36.28)
HDL-C (mg/dl) (n=165)	49.72 (10.46)	50.85 (10.63)	48.49 (10.19)
LDL-C (mg/dl) (n=164)	117.45 (29.50)	116.87 (29.62)	118.08 (29.55)
Triglycerides (mg/dl) (n=166)	147.01(129.49)	130.77 (58.26)	164.26 (129.49)
PA (REGICOR questionnaire, min/day)			
LPA (min/day)	23.60 (39.50)	24.81 (39.04)	22.30 (40.18)
MVPA (min/day)	32.81 (44.30)	35.93 (48.94)	29.46 (38.73)
Total PA (min/day)	56.41 (54.41)	60.75 (57.49)	51.76 (50.85)
SB (NHS questionnaire) (n, [%])			
No sedendary behaviour (<7h/day sitted)	118 (67.82)	66 (73.33)	52 (61.90)
Sedendary behaviour (≥7h/day sitted)	56 (32.18)	24 (26.67)	32 (38.10)
Total TV viewing (h/day)	3.26 (1.90)	3.33 (1.90)	3.19 (1.92)
Total sitting time (h/day)	5.84 (1.89)	5.65 (1.75)	6.04 (2.02)
MedDiet adherence			
MedDiet score (14 ítems)	7.05 (2.01)	7.24 (2.02)	6.83 (1.99)
High MedDiet adherence (n, [%])	76 (43.68)	42 (46.67)	34 (40.48)
Low MedDiet adherence (n, [%])	98 (56.32)	48 (53.33)	50 (59.52)

Data are presented as the mean and standard deviation for continuous variables, and as the number of participants and % for categorical variables. BMI, Body Mass Index; WC, Waist Circumference; SBP, Systolic Blood Pressure; DBP Diastolic Blood Pressure; HbA1c, Hemoglobin A1c; FPG, Fasting Plasma Glucose; HDL-C, High Density Lipoprotein Cholesterol; LDL-C, Low Density Lipoprotein Cholesterol; LPA, Light Physical Activity; MVPA, Moderate to Vigourous Physical Activity; Total PA, Total Physical Activity. *Abdominal obesity was defined as a waist circumference value of >88cm for women and >102cm for men.

### Body composition outcomes

**Table 2** shows the effects of the intervention on body composition outcomes, adjusted for potential confounders. At 4 months (mid-intervention), the IG showed a significant reduction in VAT compared to the CG (β = −0.57 [95% CI: -1.02 to -0.12], p-value= 0.013). However, this difference was not sustained at 9 months or at 15 months.

**Table 2:** Effect of the intervention on body composition at 4, 9, and 15 months.

Outcome variables	4-month			9-month			15-month		
	β	CI 95	*p-value*	β	CI 95	*p-value*	β	CI 95	*p-value*
VAT (units)	-0.57	-1.02; -0.12	<0.01	-0.32	-0.87; 0.24	0.26	0.36	-0.18; 0.90	0.19
Muscle mass (kg)	0.02	-0.60; 0.63	0.96	0.41	-1.42; 0.60	0.42	-0.22	-0.87; 0.43	0.51
Body fat mass (kg)	-0.96	-1.76; -0.15	0.02	-1.15	-2.27; -0.03	0.04	0.10	-0.94; 1.20	0.81
BMI (kg/m^2^)	-0.28	-0.57; 0.02	0.06	-0.24	-0.63; 0.16	0.24	0.16	-0.28; 0.59	0.49
WC (cm)	-1.97	-3.38; -0.56	<0.01	-1.70	-3.16; -0.23	0.02	0.95	-1.68; 1.87	0.11

The values show the *β* coefficients (95% CIs). These represent the change in outcome variables (muscle mass mass [kg], body fat mass [%] and body fat mass [kg]) due to the intervention at 4-, 9-, and 15-month follow-up. Adjusted for each outcome variable at baseline, age, and sex. VAT, Visceral Adipose Tissue; BMI, Body Mass Index; WC, Waist Circumference; CI, Confidence Interval.

Similarly, total fat mass was significantly reduced in the IG as compared to the CG at 4 months (β = −0.96 [95% CI: -1.76 to -0.15], p-value=0.02). This difference persisted at 9 months (β = −1.15 [95% CI: -2.27 to -0.03], p-value=0.04), but not at 15 months.

Waist circumference was also significantly reduced in the IG at 4 months (β = −1.97 [95% CI: -3.38 to -0.56], p-value= <0.01) and 9 months (β = −1.70 [95% CI: -3.16 to -0.23], p-value=0.02), with no sustained effects at 15 months.

No significant between-group differences were observed for muscle mass at any time point. Changes in BMI showed a similar pattern, with a non-significant reduction at 4 months and no differences at later follow-ups.

Sex-stratified analysis (**Figure 2**) showed a significant reduction in VAT among men at 4 months (β = -1.02, p 0.009), whereas changes in women were smaller and not statistically significant. No consistent sex-specific effects were observed for muscle mass. Patterns for fat mass, BMI, and waist circumference were broadly similar between sexes, with early improvements that attenuated over time.

**Figure 2:**
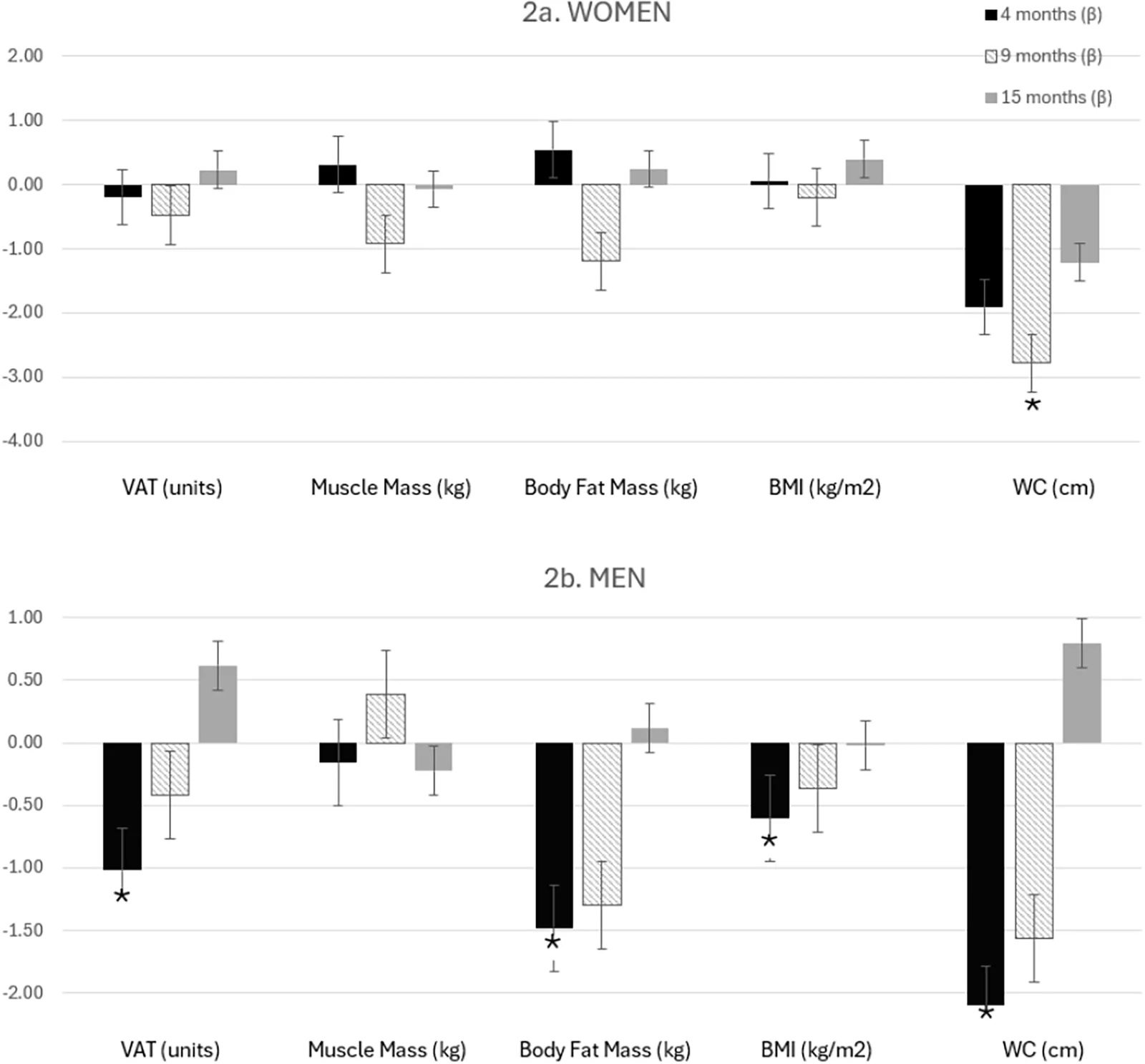
Effect of the intervention on body composition at 4, 9, and 15 months stratified by sex, **(A)** women and **(B)** men. Linear regression of VAT, muscle mass (kg), body fat mass (kg) BMI (kg/m^2^), and WC (cm) (for the intervention group at each visit (4, 9, and 15 months), adjusted for age, and each outcome variable at baseline, stratified by sex. VAT, Visceral Adipose Tissue, BMI, Body Mass Index; WC, Waist Circumference.

### Intermediate behavioral outcomes

There were no significant statistical differences between groups at any time point in PA. Light PA (LPA) levels fluctuated over time in both groups, with a slight increase in the IG (from 21.15 minutes/day at baseline to 25.08 minutes/day at 15 months), and a decrease in the CG (from 24.81 minutes/day at baseline to 21.47 minutes/day at the 15 months). Moderate-to-vigorous physical activity (MVPA) increased in both groups during follow-up; however, these changes were similar between groups (**Supplementary Appendix 1**, **Supplementary Table 2**).

In contrast, adherence to MedDiet improved significantly in the IG throughout the study period. Adherence scores in the IG increased from 6.83 (SD 1.99) at baseline to 8.29 (SD 2.18) at 4 months (p-value= <.01), 8.39 (SD 2.01) at 9 months (p-value= <.01), and 8.48 (SD 2.21) at 15 months (p-value= <.01). In contrast, the CG showed only minor changes over time, with no statistically significant differences across time points (**Supplementary Appendix 1**, **Supplementary Table 3**).

### Additional observations

No adverse effects to the intervention were observed during the study.

## Discussion

In this secondary analysis of the PREDIPHONE randomized controlled trial, a low-intensity, nurse-led telephone lifestyle intervention produced statistically significant but modest short-term reductions in selected body composition outcomes among individuals with prediabetes and overweight or obesity. Specifically, the intervention significantly reduced VAT, total fat mass, and WC at 4 months and, for some outcomes, these reductions were maintained at 9 months. However, these effects were not sustained at 15 months, and no significant changes were observed in muscle mass nor BMI at any time point compared with an active SMS-based lifestyle intervention.

The present results are consistent with previous research suggesting that lifestyle interventions can achieve short-term improvements in body composition, but that maintaining these improvements beyond the intervention period remains difficult (27). This highlights the complexity of maintaining weight and fat loss over the long term, particularly when interventions are low in intensity (28). In this context, the loss of effect observed at 15 months in the PREDIPHONE study may be attributable to its low-intensity design, which lacked those essential components commonly associated with successful long-term lifestyle interventions (frequent contact, in-person or group delivery, or community-driven approaches, among others) and that are critical for participant engagement and achieving significant behavioral changes in the long-term (15).

Importantly, although the short-term changes observed were statistically significant their magnitude was modest and their clinical relevance was limited. To achieve clinically meaningful improvements in cardiometabolic risk factors, including FPG or HbA1c, VAT reduction typically needs to exceed 10% and be sustained over time (29, 30). The VAT reduction observed in the present analysis did not reach this clinically significant threshold, nor was it sustained long enough to produce any improvement in glycemic control.

Few randomized trials have evaluated low-intensity nurse-led or remotely delivered lifestyle interventions in people at high risk of diabetes. Overall, previous studies have reported modest effects on glycaemic control, body weight, and other anthropometric outcomes (31, 32). These findings are broadly consistent with the results observed in PREDIPHONE and suggest that low-intensity interventions with limited professional contact may have a limited impact on weight and metabolic outcomes (15). Differences between studies may be partly explained by variations in population characteristics, intervention intensity, and the lifestyle strategies used. Furthermore, the regular SMS-based lifestyle advice received by the PREDIPHONE control group may have reduced the magnitude of the between-group differences.

Moreover, the absence of changes in muscle mass further supports the limited physiological impact of this low-intensity intervention. An increase in muscle mass is desirable, as it is associated with greater insulin sensitivity and improved glucose regulation, particularly in people with prediabetes (33). In this study, participants reported low levels of engagement in MVPA, a factor that may have limited both the development of muscle mass and the sustainability of fat loss (34).

Given the established association between MVPA and improvements in VAT and lean mass (10), this limited engagement in higher-intensity PA among participants could explain the lack of sustained effects observed. High-intensity exercise modalities, such as high-intensity interval training (HIIT) or intensive structured resistance training, have been shown to reduce VAT and body fat, and to increase muscle mass (35). They also promote metabolic and cardiovascular health. In contrast, lower-intensity PA may result in more modest and transient changes in body composition (30). Although participants in the intervention group showed some early modest improvements in body composition despite reporting low levels of MVPA, these changes were not sustained, suggesting that greater intensity or structured exercise components may be required for long-term impact.

Previous literature consistently shows that higher-intensity PA interventions, particularly those involving MVPA and structured resistance training, lead to more substantial and clinically significant reductions in VAT and body fat percentage, as well as greater increases in muscle mass, when compared to lower-intensity PA (30). Taylor et al. reported that both HIIT and moderate-intensity continuous training over 3 months led to VAT reductions that persisted up to 12 months (36). In the present study, most participants engaged primarily in LPA, which may have reduced the intervention’s impact on long-term significant improvements in key body composition outcomes. These findings are in line with a previous analysis indicating that MVPA is necessary to achieve significant changes in body composition (37). Moreover, the high levels of sedentary behavior observed, combined with the difficulty in motivating participants to adopt more active lifestyles, particularly increasing MVPA, further limited the intervention’s effectiveness.

As for diet quality, there were significant differences between groups during the intervention period as well as at 15-month follow-up, as the intervention significantly improved adherence to the MedDiet; however, these improvements were not sufficient to produce sustained changes in VAT or other clinical parameters (15). This lack of effect may be partly explained by the absence of specific dietary prescriptions or structured monitoring within the intervention.

In contrast, previous studies report that targeted dietary interventions can lead to improved body composition. A 3-month dietary intervention in adults with overweight or obesity resulted in a ≥5% VAT reduction, which correlated with reductions in WC, increased HDL-c, and reduced TG (38). Higher-protein hypocaloric diets in women with overweight and obesity have been found to preserve lean mass more effectively than standard-protein diets, with differences of 1.5 kg vs 2.8 kg in lean mass loss during weight reduction phases (39). Moreover, energy-restricted Mediterranean-style diets, including polyphenol-rich variations, have demonstrated significant reductions in total and visceral fat while attenuating lean mass loss (40).

In addition, interventions combining diet and PA show even more robust and sustained outcomes, as they consistently demonstrate modest yet clinically meaningful weight loss within the first six months (5–9%), along with the prevention of weight regain for up to 24 months (41). Importantly, these changes are often accompanied by a parallel reduction in VAT (28).

It is worth mentioning that when these interventions incorporate behavior change techniques, including goal setting, motivational interviewing, and tailored feedback, as well as personalized exercise prescriptions and culturally appropriate dietary guidance, they can improve both short and long-term adherence, potentially leading to sustainable improvements in body composition and metabolic health (42, 43). Although our intervention incorporated evidence-based behavior change techniques [the 5A’s model (44)], their effectiveness may depend on the intensity, duration, and context of delivery. In the present study, while improvements in dietary behavior were sustained, changes in physical activity were limited, suggesting that additional continuous support or more structured components may be required to achieve and maintain more demanding behavioral changes.

Personalization is a recognized key component of effective lifestyle interventions, particularly among populations with metabolic risk factors such as prediabetes and obesity (42). However, tailoring interventions to individuals’ metabolic profile and physical fitness level is not sufficient. The psychosocial context should also be considered to enhance participant engagement and adherence (42). In the present study, participants were predominantly from low socioeconomic and educational backgrounds, which are known to be associated with multiple challenges that can negatively impact adherence. These include financial limitations, lower health literacy, time constraints, and limited access to healthy food options and safe environments for PA (45). Consequently, it is necessary to tailor interventions that address these structural and contextual barriers (46). Although the telephone-based delivery provided an accessible and scalable approach (45), these strategies alone may not be sufficient to overcome broader contextual challenges.

Future interventions could be enhanced by integrating community-based resources, developing materials tailored to varying literacy levels, and providing flexible delivery formats that accommodate competing work and family responsibilities (46). Assessing the long-term effectiveness and feasibility of such approaches remains a key research priority for reducing health disparities associated with obesity and metabolic disease (46).

Overall, these findings suggest that a low-intensity lifestyle intervention can initiate improvements in body composition but has reduced capacity to generate sustained long-term improvements among individuals with prediabetes and overweight or obesity. To achieve clinically meaningful and durable effects, future interventions should integrate structured and progressive physical activity components, particularly resistance training and higher-intensity modalities, alongside targeted dietary strategies (9).

Moreover, future research should also explore how such higher-intensity interventions can be feasibly and effectively adapted to the specific needs and barriers of individuals with low socioeconomic and educational backgrounds, prediabetes, obesity and/or metabolic disorders, who might initially have limited capacity or motivation for high-intensity exercise (47).

At the same time, to further improve effectiveness and accessibility, a teamwork approach between healthcare providers and PA professionals is necessary. These partnerships could facilitate the development of safe and personalized programs for people with chronic conditions (48). Furthermore, community-based programs tend to succeed when facilities are perceived as inclusive, welcoming, and staffed by qualified professionals (49).

### Limitations and strengths

The present study has some limitations. First, as a secondary outcomes analysis of the PREDIPHONE trial, it was not specifically powered to detect changes in body composition outcomes. Although the findings provide relevant information regarding the effects of the intervention on BMI and fat mass, the sample size was not calculated for these outcomes. Therefore, the lack of statistical significance in some results might reflect limited power due to the small sample size rather than a lack of a significant effect.

Second, body composition was assessed by BIA, which is influenced by factors such as hydration status and fluid balance (50). To mitigate these limitations, all BIA assessments were performed under standardized conditions. Moreover, despite its sensitivity to hydration, BIA provides a more objective and reproducible measure of body composition—including VAT—than traditional anthropometric techniques.

Third, the use of self-reported measures for physical activity and dietary intake may have introduced recall or social desirability bias.

Additionally, the study population consisted predominantly of individuals with prediabetes and lower socioeconomic and educational levels. This might limit generalizability to younger populations or different healthcare settings but also reflects a high-risk group for whom effective interventions are particularly needed (51).

Furthermore, as the intervention was of low intensity, it may not have been sufficiently intensive to produce all anticipated medium- and long-term body composition changes. In parallel, participant adherence to this type of intervention represents a pivotal determinant when evaluating clinical effectiveness.

The COVID-19 pandemic may have also influenced participant involvement and intervention delivery.

Finally, separate general linear models were fitted at each time point rather than a repeated-measures framework. Although this approach was pre-specified and is consistent with the analytical strategy of the primary analysis, it does not explicitly model within-participant correlation across time points and does not allow formal testing of whether the intervention effect changes over time.

PREDIPHONE incorporated a person-centred approach aimed at promoting sustainable lifestyle changes; however, achieving clinically meaningful and sustained changes in body composition may require greater intervention intensity and longer-term support. Future research should therefore include implementation studies to determine how effective lifestyle interventions can be delivered and maintained in real-world settings. Co-design approaches involving the target population and relevant healthcare stakeholders may further improve the acceptability, feasibility, and long-term sustainability of these interventions by ensuring that recommendations are aligned with individual needs and the practical context in which lifestyle changes take place.

A key strength of the study is its pragmatic design, including the use of an active control group, which enhances external validity and reflects real-world primary care conditions. The relatively high follow-up rate further supports the robustness of the findings.

## Conclusions

In a population with prediabetes and overweight or obesity, a low-intensity, nurse-led telephone lifestyle intervention showed short-term improvements in VAT and total fat mass at 4- and 9-month follow-up. However, these benefits were not sustained post-intervention, and no significant changes in muscle mass were observed throughout the intervention period.

These results highlight the need for future interventions to focus not only on initial improvements on body composition parameters, but also on sustaining these changes, to achieve clinically relevant changes in adults with prediabetes. While phone-based interventions may serve as an accessible and effective entry point for initiating lifestyle changes, they are insufficient as a standalone strategy to achieve and maintain clinically meaningful improvements in body composition. Incorporating sustained approaches that extend beyond individual counseling and integrate group support or community-based strategies may enhance engagement, adherence and maintenance, and improve long-term outcomes.

## Data Availability

The original contributions presented in the study are included in the article/**Supplementary Material**. Further inquiries can be directed to the corresponding author.

## References

[1] World Obesity Federation . World Obesity Atlas. London, UK: World Obesity Federation (2023).

[2] Collaborators GBDAB . Global, regional, and national prevalence of adult overweight and obesity, 1990-2021, with forecasts to 2050: a forecasting study for the Global Burden of Disease Study 2021. Lancet. (2025) 405:813–38. doi: 10.1016/S0140-6736(25)00355-1 40049186 PMC11920007

[3] Perez-Rodrigo C Hervas Barbara G Gianzo Citores M Aranceta-Bartrina J . Prevalence of obesity and associated cardiovascular risk factors in the Spanish population: the ENPE study. Rev Esp Cardiol (Engl Ed). (2022) 75:232–41. doi: 10.1016/j.rec.2020.12.020 33773941

[4] Egan AM Wood-Wentz CM Mohan S Bailey KR Vella A . Baseline fasting glucose level, age, sex, and body mass index and the development of diabetes in US adults. JAMA Netw Open. (2025) 8:e2456067. doi: 10.1001/jamanetworkopen.2024.56067 39847352 PMC11758592

[5] Kanbour S Ageeb RA Malik RA Abu-Raddad LJ . Impact of bodyweight loss on type 2 diabetes remission: a systematic review and meta-regression analysis of randomised controlled trials. Lancet Diabetes Endocrinol. (2025) 13:294–306. doi: 10.1016/S2213-8587(24)00346-2 40023186

[6] Konieczna J Abete I Galmes AM Babio N Colom A Zulet MA . Body adiposity indicators and cardiometabolic risk: Cross-sectional analysis in participants from the PREDIMED-Plus trial. Clin Nutr. (2019) 38:1883–91. doi: 10.1016/j.clnu.2018.07.005 30031660

[7] American Diabetes Association Professional Practice C . 3. Prevention or delay of diabetes and associated comorbidities: standards of care in diabetes-2025. Diabetes Care. (2025) 48:S50–8. doi: 10.2337/dc25-S003 39651971 PMC11635039

[8] Hemmingsen B Gimenez-Perez G Mauricio D Roque IFM Metzendorf MI Richter B . Diet, physical activity or both for prevention or delay of type 2 diabetes mellitus and its associated complications in people at increased risk of developing type 2 diabetes mellitus. Cochrane Database Syst Rev. (2017) 12:CD003054. doi: 10.1002/14651858.CD003054.pub4 29205264 PMC6486271

[9] Bennasar-Veny M Malih N Galmes-Panades AM Hernandez-Bermudez IC Garcia-Coll N Ricci-Cabello I . Effect of physical activity and different exercise modalities on glycemic control in people with prediabetes: a systematic review and meta-analysis of randomized controlled trials. Front Endocrinol (Lausanne). (2023) 14:1233312. doi: 10.3389/fendo.2023.1233312 37842295 PMC10569497

[10] Wewege MA Desai I Honey C Coorie B Jones MD Clifford BK . The effect of resistance training in healthy adults on body fat percentage, fat mass and visceral fat: A systematic review and meta-analysis. Sports Med. (2022) 52:287–300. doi: 10.1007/s40279-021-01562-2 34536199

[11] Salas-Salvado J Bullo M Estruch R Ros E Covas MI Ibarrola-Jurado N . Prevention of diabetes with Mediterranean diets: a subgroup analysis of a randomized trial. Ann Intern Med. (2014) 160:1–10. doi: 10.7326/M13-1725 24573661

[12] Galaviz KI Weber MB Straus A Haw JS Narayan KMV Ali MK . Global diabetes prevention interventions: A systematic review and network meta-analysis of the real-world impact on incidence, weight, and glucose. Diabetes Care. (2018) 41:1526–34. doi: 10.2337/dc17-2222 29934481 PMC6463613

[13] Golovaty I Ritchie ND Tuomilehto J Mohan V Ali MK Gregg EW . Two decades of diabetes prevention efforts: A call to innovate and revitalize our approach to lifestyle change. Diabetes Res Clin Pract. (2023) 198:110195. doi: 10.1016/j.diabres.2022.110195 36470316 PMC10079599

[14] Abbate M Fresneda S Yanez A Ricci-Cabello I Galmes-Panades AM Aguilo A . Nurse-led telephone intervention for lifestyle changes on glycaemic control in people with prediabetes: Study protocol for a randomized controlled trial. J Adv Nurs. (2021) 77:3204–17. doi: 10.1111/jan.14842 33769603

[15] Arias-Fernandez M Huguet-Torres A Abbate M Fresneda S Torres-Carballo M Carvalho-Azevedo A . Effectiveness of a low-intensity nurse-led lifestyle intervention on glycaemic control in individuals with prediabetes: The PREDIPHONE randomized controlled clinical trial. Int J Nurs Stud. (2025) 165:105034. doi: 10.1016/j.ijnurstu.2025.105034 40058011

[16] American Diabetes Association Professional Practice C . 2. Diagnosis and classification of diabetes: standards of care in diabetes-2025. Diabetes Care. (2025) 48:S27–49. doi: 10.2337/dc25-S002 39651986 PMC11635041

[17] Domingo-Salvany A Bacigalupe A Carrasco JM Espelt A Ferrando J Borrell C . Proposals for social class classification based on the Spanish National Classification of Occupations 2011 using neo-Weberian and neo-Marxist approaches. Gac Sanit. (2013) 27:263–72. doi: 10.1016/j.gaceta.2012.12.009 23394892

[18] WHO . Guidelines for Controlling and Monitoring the Tobacco Epidemic. Geneva, Switzerland: WHO (1998).

[19] Stewart A Marfell-Jones M Olds T De Ridder J . International Standars for Anthropometric Assessment, 2019: International Society for the Advancement of Kinanthropometry (Isak). (2019) 137.

[20] Kelly J Metcalfe J . Validity and reliability of body composition analysis using the tanita BC418-MA. J Exercise Physiol Online. (2012) 15:74–83.

[21] Salas-Salvado J Rubio MA Barbany M Moreno B Grupo Colaborativo de la S . SEEDO 2007 Consensus for the evaluation of overweight and obesity and the establishment of therapeutic intervention criteria. Med Clin (Barc). (2007) 128:184–96. doi: 10.1016/s0025-7753(07)72531-9 17298782

[22] Gorostidi M Gijon-Conde T de la Sierra A Rodilla E Rubio E Vinyoles E . 2022 Practice guidelines for the management of arterial hypertension of the Spanish Society of Hypertension. Hipertens Riesgo Vasc. (2022) 39:174–94. doi: 10.1016/j.hipert.2022.09.002 36153303

[23] Molina L Sarmiento M Penafiel J Donaire D Garcia-Aymerich J Gomez M . Validation of the regicor short physical activity questionnaire for the adult population. PloS One. (2017) 12:e0168148. doi: 10.1371/journal.pone.0168148 28085886 PMC5234797

[24] Ainsworth BE Haskell WL Herrmann SD Meckes N Bassett DR Jr. Tudor-Locke C . 2011 Compendium of Physical Activities: a second update of codes and MET values. Med Sci Sports Exerc. (2011) 43:1575–81. doi: 10.1249/MSS.0b013e31821ece12 21681120

[25] Martinez-Gonzalez MA Lopez-Fontana C Varo JJ Sanchez-Villegas A Martinez JA . Validation of the Spanish version of the physical activity questionnaire used in the Nurses' Health Study and the Health Professionals' Follow-up Study. Public Health Nutr. (2005) 8:920–7. doi: 10.1079/phn2005745 16277809

[26] Martinez-Gonzalez MA Garcia-Arellano A Toledo E Salas-Salvado J Buil-Cosiales P Corella D . A 14-item Mediterranean diet assessment tool and obesity indexes among high-risk subjects: the PREDIMED trial. PloS One. (2012) 7:e43134. doi: 10.1371/journal.pone.0043134 22905215 PMC3419206

[27] Franz MJ Boucher JL Rutten-Ramos S VanWormer JJ . Lifestyle weight-loss intervention outcomes in overweight and obese adults with type 2 diabetes: a systematic review and meta-analysis of randomized clinical trials. J Acad Nutr Diet. (2015) 115:1447–63. doi: 10.1016/j.jand.2015.02.031 25935570

[28] Franz MJ VanWormer JJ Crain AL Boucher JL Histon T Caplan W . Weight-loss outcomes: a systematic review and meta-analysis of weight-loss clinical trials with a minimum 1-year follow-up. J Am Diet Assoc. (2007) 107:1755–67. doi: 10.1016/j.jada.2007.07.017 17904936

[29] Nicklas BJ Penninx BW Ryan AS Berman DM Lynch NA Dennis KE . Visceral adipose tissue cutoffs associated with metabolic risk factors for coronary heart disease in women. Diabetes Care. (2003) 26:1413–20. doi: 10.2337/diacare.26.5.1413 12716798

[30] Jakicic JM Rogers RJ Davis KK Collins KA . Role of physical activity and exercise in treating patients with overweight and obesity. Clin Chem. (2018) 64:99–107. doi: 10.1373/clinchem.2017.272443 29158251

[31] Hesselink AE Rutten GE Slootmaker SM de Weerdt I Raaijmakers LG Jonkers R . Effects of a lifestyle program in subjects with Impaired Fasting Glucose, a pragmatic cluster-randomized controlled trial. BMC Fam Pract. (2015) 16:183. doi: 10.1186/s12875-015-0394-7 26695176 PMC4688978

[32] Mira-Martinez S Malih N Angullo-Martinez E Zamanillo-Campos R Yanez AM Bennasar-Veny M . A multifaceted digital intervention for the prevention of type 2 diabetes mellitus in primary care (PREDIABETEXT): cluster randomized trial. J Med Internet Res. (2025) 27:e70981. doi: 10.2196/70981 41067715 PMC12550449

[33] Srikanthan P Karlamangla AS . Relative muscle mass is inversely associated with insulin resistance and prediabetes. Findings from the third National Health and Nutrition Examination Survey. J Clin Endocrinol Metab. (2011) 96:2898–903. doi: 10.1210/jc.2011-0435 21778224

[34] Rosique-Esteban N Babio N Diaz-Lopez A Romaguera D Alfredo Martinez J Sanchez VM . Leisure-time physical activity at moderate and high intensity is associated with parameters of body composition, muscle strength and sarcopenia in aged adults with obesity and metabolic syndrome from the PREDIMED-Plus study. Clin Nutr. (2019) 38:1324–31. doi: 10.1016/j.clnu.2018.05.023 29910068

[35] Schwingshackl L Dias S Strasser B Hoffmann G . Impact of different training modalities on anthropometric and metabolic characteristics in overweight/obese subjects: a systematic review and network meta-analysis. PloS One. (2013) 8:e82853. doi: 10.1371/journal.pone.0082853 24358230 PMC3866267

[36] Taylor JL Holland DJ Mielke GI Bailey TG Johnson NA Leveritt MD . Effect of high-intensity interval training on visceral and liver fat in cardiac rehabilitation: A randomized controlled trial. Obes (Silver Spring). (2020) 28:1245–53. doi: 10.1002/oby.22833 32475048

[37] Torres-Carballo M Galmes-Panades AM Arias-Fernandez M Huguet-Torres A Abbate M Fresneda S . Isotemporal substitution of sedentary time with physical activity and sleeping time: associations with body composition among individuals with prediabetes. Front Sports Act Living. (2025) 7:1579962. doi: 10.3389/fspor.2025.1579962 40260421 PMC12009759

[38] Abdullah SR Nur Zati Iwani AK Ahmad Zamri L Wan Mohd Zin RM Abu Seman N Zainal Abidin NA . Visceral adiposity loss is associated with improvement in cardiometabolic markers: findings from a dietary intervention study. Front Endocrinol (Lausanne). (2025) 16:1576599. doi: 10.3389/fendo.2025.1576599 40535330 PMC12173930

[39] Leidy HJ Carnell NS Mattes RD Campbell WW . Higher protein intake preserves lean mass and satiety with weight loss in pre-obese and obese women. Obes (Silver Spring). (2007) 15:421–9. doi: 10.1038/oby.2007.531 17299116

[40] Konieczna J Ruiz-Canela M Galmes-Panades AM Abete I Babio N Fiol M . An energy-reduced mediterranean diet, physical activity, and body composition: an interim subgroup analysis of the PREDIMED-plus randomized clinical trial. JAMA Netw Open. (2023) 6:e2337994. doi: 10.1001/jamanetworkopen.2023.37994 37851444 PMC10585413

[41] Dombrowski SU Knittle K Avenell A Araujo-Soares V Sniehotta FF . Long term maintenance of weight loss with non-surgical interventions in obese adults: systematic review and meta-analyses of randomised controlled trials. BMJ. (2014) 348:g2646. doi: 10.1136/bmj.g2646 25134100 PMC4020585

[42] Zeevi D Korem T Zmora N Israeli D Rothschild D Weinberger A . Personalized nutrition by prediction of glycemic responses. Cell. (2015) 163:1079–84. doi: 10.1016/j.cell.2015.11.001 26590418

[43] Group TDPPR . The Diabetes Prevention Program (DPP): Description of lifestyle intervention. Diabetes Care. (2002) 25:2165–71. doi: 10.2337/diacare.25.12.2165 12453955 PMC1282458

[44] Glasgow RE Funnell MM Bonomi AE Davis C Beckham V Wagner EH . Self-management aspects of the improving chronic illness care breakthrough series: implementation with diabetes and heart failure teams. Ann Behav Med. (2002) 24:80–7. doi: 10.1207/S15324796ABM2402_04 12054323

[45] Gittelsohn J Rowan M . Preventing diabetes and obesity in American Indian communities: the potential of environmental interventions. Am J Clin Nutr. (2011) 93:1179S–83S. doi: 10.3945/ajcn.110.003509 21411614

[46] Kumanyika SK Whitt-Glover MC Haire-Joshu D . What works for obesity prevention and treatment in black Americans? Research directions. Obes Rev. (2014) 15:204–12. doi: 10.1111/obr.12213 25196414

[47] Candib LM Silva M Cashman SB Ellstrom D Mallett K . Creating open access to exercise for low-income patients through a community collaboration for quality improvement: if you build it, they will come. J Ambul Care Manage. (2008) 31:142–50. doi: 10.1097/01.JAC.0000314704.34795.8a 18360175

[48] Bird ML Mortenson BW Chu F Acerra N Bagnall E Wright A . Building a bridge to the community: an integrated knowledge translation approach to improving participation in community-based exercise for people after stroke. Phys Ther. (2019) 99:286–96. doi: 10.1093/ptj/pzy146 30698783 PMC6383711

[49] Reed JL Harris JM Midence L Yee EB Grace SL . Evaluating the Heart Wise Exercise program: a model for safe community exercise programming. BMC Public Health. (2016) 16:190–8. doi: 10.1186/s12889-016-2866-7 26922240 PMC4769838

[50] O'Brien C Young AJ Sawka MN . Bioelectrical impedance to estimate changes in hydration status. Int J Sports Med. (2002) 23:361–6. doi: 10.1055/s-2002-33145 12165888

[51] Carvalho-Azevedo A Abbate M Fresneda S Arias-Fernandez M Torres-Carballo M Yanez AM . Influence of social determinants on lifestyle modification adherence in people with prediabetes: a sequential explanatory mixed-methods study protocol. Front Public Health. (2025) 13:1591205. doi: 10.3389/fpubh.2025.1591205 40678653 PMC12267270

