# Supplementary material for: Effectiveness of a nurse-led phone intervention on body composition in adults with prediabetes and overweight/obesity: PREDIPHONE secondary analysis
Source: Front Endocrinol (Lausanne). 2026 Sep 17;17:1911675. doi: 10.3389/fendo.2026.1911675 (PMC13627063; doi:10.3389/fendo.2026.1911675)
Supplement: Supplementary file 1 [file Table1.docx]

### Supplementary Material

| **Supplementary Table 1. Baseline characteristics of participants according to study completion status.** | | | |  |  |  |
| --- | --- | --- | --- | --- | --- | --- |
| **Variables** | **Total (n=174)** | **Complete the intervention (n=161)** | **Lost to follow-up at 15^th^ month (n=13)** |  |  |  |
|  |  |  |  |  |  |  |
| **Age** | 61.10 (8.90) | 61.3 (8.7) | 59.2 (11.5) |  |  |  |
|  |  |  |  |  |  |  |
| **Woman (n, [%])** | 91 (52.30) | 83 (51.6) | 8 (61.5) |  |  |  |
|  | | | |  |  |  |
| **Social class^1^ (n, [%]):** | | | |  |  |  |
|  | | | |  |  |  |
| White collar | 35 (20.12) | 33 (20.5) | 2 (15,4) |  |  |  |
| Blue collar | 139 (79.88) | 128 (79.5) | 11 (84,6) |  |  |  |
|  | | | |  |  |  |
| **Education level (n, [%]):** | | | |  |  |  |
|  | | | |  |  |  |
| University degree | 29 (16.67) | 27 (16.8) | 2 (15.4) |  |  |  |
| Higher education | 60 (34.48) | 57 (35.4) | 3 (23.1) |  |  |  |
| Basic studies | 85 (48.85) | 77 (47.8) | 8 (61,6) |  |  |  |
|  | | | |  |  |  |
| **Smoking habits^2^ (n, [%]):** | | | |  |  |  |
|  | | | |  |  |  |
| Never | 78 (44.83) | 71 (44.1) | 7 (53,8) |  |  |  |
| Current | 22 (12.64) | 19 (11.8) | 3 (23.1) |  |  |  |
| Former | 74 (42.53) | 71 (44.1) | 3 (23.1) |  |  |  |
|  | | | |  |  |  |
| **Anthropometric measures:** | | | |  |  |  |
|  | | | |  |  |  |
| BMI (kg/m^2^) | 32.17 (3.46) | 32.2 (3.5) | 31.8 (3.3) |  |  |  |
| WC (cm) | 105.10 (10.32) | 105.2 (10.4) | 103.9 (9.3) |  |  |  |
| Abdominal obesity^a^ |  |  |  |  |  |  |
| No | 25 (14.37) | 23 (14.3) | 2 (15.4) |  |  |  |
| Yes | 149 (85.63) | 138 (85.7) | 11 (84.6) |  |  |  |
|  | | | |  |  |  |
| **Body composition:** | | | |  |  |  |
|  | | | |  |  |  |
| VAT (units) | 13.50 (3.94) | 13.5 (4.0) | 12.8 (3.4) |  |  |  |
| Body fat mass (kg) | 30.71 (7.96) | 30.8 (8.1) | 29.1 (6.8) |  |  |  |
| Body fat mass (%) | 36.00 (6.96) | 36.0 (6.9) | 35.6 (7.8) |  |  |  |
| Lean body mass (kg) | 54.63 (11.32) | 54.7 (11.3) | 53.3 (11.4) |  |  |  |
|  | | | |  |  |  |
| **Clinical parameters:** | | | |  |  |  |
|  | | | |  |  |  |
| SBP (mmHg) | 134.92 (14.65) | 135.3 (14.9) | 130.1 (10.7) |  |  |  |
| DBP (mmHg) | 84.05 (9.31) | 84.2 (9.4) | 82.6 (7.7) |  |  |  |
| HbA1c (%) (n=135) | 5.90 (0.33) | 5.9 (0.3) | 5.9 (0.3) |  |  |  |
| FPG (mg/dl) | 108.98 (6.47) | 109.1 (6.4) | 107.2 (7.0) |  |  |  |
| Total cholesterol (mg/dl) (n=168) | 195.45 (34.95) | 196.5 (35.5) | 182.4 (24.8) |  |  |  |
| HDL-C (mg/dl) (n=165) | 49.72 (10.46) | 49.7 (10.5) | 50.2 (10.3) |  |  |  |
| LDL-C (mg/dl) (n=164) | 117.45 (29.50) | 117.9 (29.9) | 112.3 (24.4) |  |  |  |
| Triglycerides (mg/dl) (n=166) | 147.01(129.49) | 141.5 (61.6) | 99.3 (44.7) |  |  |  |
|  | | | |  |  |  |
| **PA (REGICOR questionnaire, min/day)** | | | |  |  |  |
|  | | | |  |  |  |
| LPA (min/day) | 23.60 (39.50) | 23.3 (40.5) | 27.5 (25.3) |  |  |  |
| MVPA (min/day) | 32.81 (44.30) | 33.8 (45.4) | 20.3 (24.1) |  |  |  |
| Total PA (min/day) | 56.41 (54.41) | 57.1 (55.8) | 47.7 (33.8) |  |  |  |
|  | | | |  |  |  |
| **SB (NHS questionnaire) (n, [%])** | | | |  |  |  |
|  | | | |  |  |  |
| No sedendary behaviour (<7h/day sitted) | 118 (67.82) | 109 (67.7) | 9 (69.2) |  |  |  |
| Sedendary behaviour (≥7h/day sitted) | 56 (32.18) | 52 (32.3) | 4 (30.8) |  |  |  |
| Total TV viewing (h/day) | 3.26 (1.90) | 3.2 (1.9) | 3.5 (2.4) |  |  |  |
| Total sitting time (h/day) | 5.84 (1.89) | 5.9 (1.9) | 5.2 (2.0) |  |  |  |
|  |  |  |  |  |  |  |
| **MedDiet adherence** |  |  |  |  |  |  |
|  |  |  |  |  |  |  |
| MedDiet score (14 ítems) | 7.05 (2.01) | 7.0 (2.0) | 7.2 (1.5) |  |  |  |
| High MedDiet adherence (n, (%)) | 76 (43.68) | 71 (44.1) | 5 (38.5) |  |  |  |
| Low MedDiet adherence (n, (%)) | 98 (56.32) | 90 (55.9) | 8 (61.5) |  |  |  |
| Data are presented as the mean and standard deviation for continuous variables, and as the number of participants and % for categorical variables. Abbreviations: BMI, Body Mass Index; WC, Waist Circumference; SBP, Systolic Blood Pressure; DBP Diastolic Blood Pressure; HbA1c, Hemoglobin A1c; FPG, Fasting Plasma Glucose; HDL-C, High Density Lipoprotein Cholesterol; LDL-C, Low Density Lipoprotein Cholesterol; LPA, Light Physical Activity; MVPA, Moderate to Vigourous Physical Activity; Total PA, Total Physical Activity. *Abdominal obesity was defined as a waist circumference value of >88cm for women and >102cm for men | | | |  |  |  |
|  | | | |  |  |  |

**Supplementary Table 2. Physical activity in the control and intervention groups at 4, 9, and 15 months**

|  | |  | **LPA** | | | | | | | | | | |
| --- | --- | --- | --- | --- | --- | --- | --- | --- | --- | --- | --- | --- | --- |
|  | | **Baseline** | | | **4-month** | | | **9-month** | | | **15-month** | | |
|  |  | **Mean** | **SD** | **p-value** | **Mean** | **SD** | **p-value** | **Mean** | **SD** | **p-value** | **Mean** | **SD** | **p-value** |
|  | |  |  |  |  |  |  |  |  |  |  |  |  |
| **Control Group** | | 24.81 | 39.04 |  | 24.51 | 36.17 |  | 21.20 | 31.75 |  | 21.47 | 32.55 |  |
| **Intervention Group** | | 22.29 | 40.18 | .68 | 21.15 | 30.83 | .51 | 29.10 | 59.03 | .27 | 16.62 | 25.08 | .29 |
|  | |  |  |  |  |  |  |  |  |  |  |  |  |
|  | | **MVPA** | | | | | | | | | | | |
|  | | **Baseline** | | | **4-month** | | | **9-month** | | | **15-month** | | |
|  | | **Mean** | **SD** | **p-value** | **Mean** | **SD** | **p-value** | **Mean** | **SD** | **p-value** | **Mean** | **SD** | **p-value** |
|  | |  |  |  |  |  |  |  |  |  |  |  |  |
| **Control Group** | | 35.93 | 48.94 |  | 43.48 | 59.39 |  | 42.25 | 53.89 |  | 40.42 | 63.98 |  |
| **Intervention Group** | | 29.46 | 38.73 | .34 | 52.34 | 55.73 | .32 | 46.36 | 52.91 | .61 | 38.59 | 51.01 | .84 |
|  |  |  |  | | | | | | | | | | |

The values show the mean minutes per day of physical activity at baseline, 4, 9, and 15 months. These represent the changes in physical activity levels due to the effect of the intervention at each time point. Adjusted for each outcome variable at baseline, age and sex. Abbreviations: LPA, Light Physical Activity; MVPA, Moderate-to-Vigorous Physical Activity.

**Supplementary Table 3. Diet in the control and intervention groups at 4, 9, and 15 months**

|  | |  | **MedDiet questionnaire (14 items)** | | | | | | | | | | |
| --- | --- | --- | --- | --- | --- | --- | --- | --- | --- | --- | --- | --- | --- |
|  | | **Baseline** | | | **4-month** | | | **9-month** | | | **15-month** | | |
|  |  | **Mean** | **SD** | **p-value** | **Mean** | **SD** | **p-value** | **Mean** | **SD** | **p-value** | **Mean** | **SD** | **p-value** |
|  | |  |  |  |  |  |  |  |  |  |  |  |  |
| **Control Group** | | 7.24 | 2.09 |  | 7.37 | 2.15 |  | 7.53 | 2.12 |  | 7.55 | 2.09 |  |
| **Intervention Group** | | 6.83 | 1.99 | .18 | 8.29 | 2.18 | <.01 | 8.39 | 2.01 | <.01 | 8.48 | 2.21 | <.01 |

The values show the mean adherence scores to the Mediterranean diet (14-item questionnaire) at baseline, 4, 9, and 15 months. These represent the changes in dietary habits due to the effect of the intervention at each time point. Adjusted for each outcome variable at baseline, age and sex.
